# Calcinosis Cutis Associated with Chronic Sclerodermoid Graft versus Host Disease: A Case and Review of the Literature

**DOI:** 10.1155/2020/9250923

**Published:** 2020-02-27

**Authors:** Jacqueline Deen, Lisa Byrom, Ivan Robertson

**Affiliations:** Department of Dermatology, Royal Brisbane and Women's Hospital, Brisbane, Queensland, Australia

## Abstract

We present a rare case of calcinosis cutis associated with chronic sclerodermoid graft versus host disease in a 59-year-old male, 13 years following allogenic bone marrow transplantation. The etiology of calcification was thought to be dystrophic. Further research is needed to understand the link between calcinosis cutis and chronic sclerodermoid graft versus host disease to assist with selecting appropriate management for these patients.

## 1. Introduction

Calcinosis cutis is a descriptive term for the deposition of insoluble calcium salts in the dermis and subcutaneous tissue and is associated with a number of underlying disorders. The underlying cause of this condition remains unknown. Serum calcium, phosphate, and parathyroid hormone are typically normal, although there may be reduced excretion of calcium and phosphate. There are five different types based upon etiology of calcium deposition: dystrophic, metastatic, iatrogenic, idiopathic, and calciphylaxis [1]. The most common form is the dystrophic type, which results from local tissue trauma, in the setting of normal calcium homeostasis. The clinical presentation varies in severity from asymptomatic (incidental finding on radiologic imaging) to subcutaneous nodules and plaques, which may cause associated pain or functional impairment. Dystrophic calcinosis cutis is most frequently associated with autoimmune connective tissue diseases such as dermatomyositis, systemic lupus erythematosus, and systemic sclerosis. It may also occur in patients with cutaneous neoplasms, panniculitis, porphyria cutaneous tarda, genodermatoses, localized trauma, or infection [1, 2].

Metastatic calcification occurs from abnormal calcium and phosphate metabolism, and the most common underlying cause is chronic renal failure. Iatrogenic calcinosis occurs from the deposition of calcium salts in the skin secondary to medical interventions, typically intravenous calcium gluconate and calcium chloride. Idiopathic calcification occurs in the absence of an underlying tissue injury or metabolic disorder. The final subtype, calciphylaxis, is a calcific vasculopathy of small and medium-sized blood vessels in the dermis and subcutaneous tissue, usually associated with end-stage renal failure [1, 3].

We present a rare case of calcinosis cutis associated with chronic sclerodermoid graft versus host disease. To our knowledge, this is the third reported case in the literature.

## 2. Case Report

**Figure 1:**
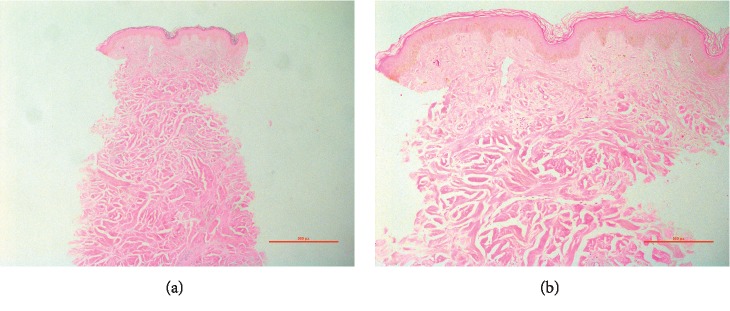
Histopathology demonstrating sclerodermoid graft versus host disease. (a), (b) A normal *epidermis* with no lichenoid reaction pattern. Within the deep reticular dermis, there are broad bundles of collagen running parallel to the skin surface.  Figure 1(b) shows mild pigment incontinence in the superficial dermis. A mild perivascular lymphocytic infiltrate is present within the deep dermis. There is no malignant infiltrate and no viral cytopathic changes or fungal organisms identified (hematoxylin and eosin staining, 1(a) original magnification × 40, 1(b) original magnification × 100).

A 59-year-old male presented for routine dermatology follow-up, 13 years following allogenic bone marrow transplantation (BMT) for grade 3 follicular non-Hodgkin lymphoma. The patient had a background history of sclerodermoid graft versus host disease (GVHD), which developed 12 months after BMT and was confirmed by cutaneous biopsy (Figure 1). He had multiorgan involvement, including the skin, eyes, oral cavity, joints, and lungs (restrictive lung disease). His GVHD was refractory to multiple systemic treatments including prednisolone, intravenous immunoglobulin (IVIG), cyclosporine, methotrexate, mycophenolate mofetil, tacrolimus, thalidomide, psoralen and ultraviolet A (PUVA), and ultraviolet A1 (UVA1).

The patient's non-Hodgkin lymphoma was diagnosed 15 years prior, and after failing chemotherapy and autologous stem cell transplantation, he underwent a BMT from an unrelated female donor with ABO compatibility. His other past medical history was significant for steroid-induced diabetes mellitus and a previous left leg deep vein thrombosis. His medications included warfarin, allopurinol, intravenous immunoglobulin, celecoxib, lubricant eye drops, prophylactic trimethoprim/sulfamethoxazole, and valaciclovir. There were no known drug allergies; the patient was a nonsmoker and worked as a pastor.

**Figure 2:**
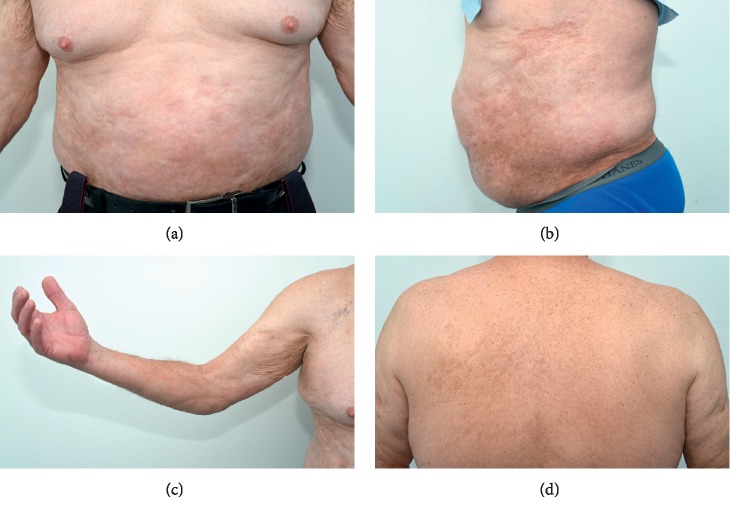
Clinical photographs showing hyperpigmented, indurated plaques of the abdomen. (a), (b) The right forearm and (c), (d) upper back.

On examination, there were indurated, woody, thickened plaques on the upper arms, forearms, and trunk, clinically consistent with sclerodermoid changes (Figure 2). There were fixed flexion deformities of the bilateral elbows and perioral skin tightening with a decreased oral aperture. As an incidental finding, there were multiple, small, nontender, stone-hard nodules palpable over the trunk and bilateral upper limbs. There was no evidence clinically of autoimmune connective tissue disease including no nail fold telangiectasia, Raynaud's disease, sclerodactyly, or a photodistributed rash. Muscle power was graded 5/5 for the upper and lower limbs.

Baseline bloods including a complete blood count, urea and electrolytes, and liver function were unremarkable, and the patient's renal function was stable (creatinine 123 *μ*mol/L, eGFR 55 mL/min). Corrected calcium, phosphate, and parathyroid hormone were within normal limits. ANA was negative.

**Figure 3:**
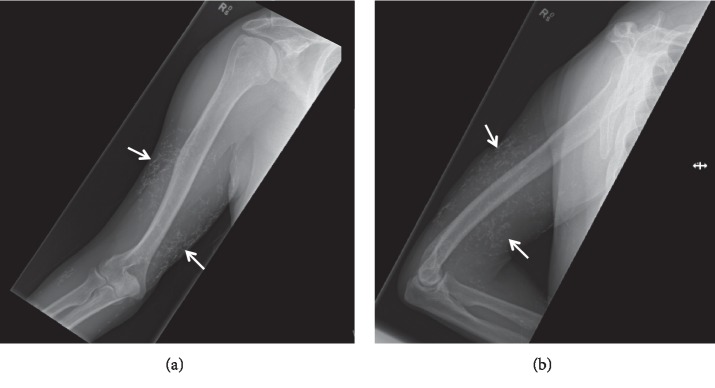
Radiographs of the anterior (a) and posterior (b) right arm and forearm show extensive linear and irregular calcifications (arrows) in the field of view. The distribution of the calcified foci includes the subcutaneous fat.

Radiographs showed diffuse calcified nodules of the soft tissue overlaying the right humerus and proximal radius and ulnar (Figure 3), indicative of calcinosis cutis. Treatment was not initiated, as the patient was asymptomatic, and there was no evidence of lesion extrusion.

## 3. Discussion

Graft versus host disease has numerous cutaneous manifestations that are traditionally divided into acute and chronic forms based on the time of onset. Acute GVHD occurs within 100 days of transplantation, while chronic forms present after 100 days [4]. However, since signs of acute and chronic GVHD can present outside of these time periods, there has been increased use of clinical findings, rather than a designated time period to differentiate between acute and chronic GVHD [5].

Chronic GVHD has an incidence of approximately 40% in BMT recipients, although this is variable (between 6 and 80%) depending upon the presence of risk factors and diagnostic criteria used. The risk factors associated with chronic GVHD are well described, including an unrelated donor, donor-recipient sex mismatch, and older age (>40 years) of the recipient at the time of the BMT [6, 7]. In a multivariate analysis by Skert et al., significant predictors for the development of skin sclerosis in chronic GVHD were defined as disorders of pigmentation, eosinophilia, and autoimmunity [8]. Our patient had many risk factors for development of GVHD and, however, did not have any risk factors for development of sclerodermoid subtype.

Calcinosis cutis associated with sclerodermoid GVHD remains an interesting yet rarely discussed topic in the literature (Table 1) [9, 10]. In the two previously published cases and in our patient, the patient age was greater than 50 years, serum calcium was normal, the time interval between the initial diagnosis of sclerodermoid GVHD and the development of calcinosis cutis was greater than 10 years (average 11.5 years), and the aetiology of calcification was thought to be dystrophic, related to chronic sclerodermoid change. Furthermore, the distribution of calcinosis cutis involved tissues affected by sclerodermatous changes; thus, it is likely that the calcification is secondary to tissue injury related to chronic GVHD.

Treatment of calcinosis cutis is difficult as data on efficacy is limited and conflicting. The literature is mainly based on treatments for dystrophic calcinosis in patients with autoimmune conditions such as dermatomyositis and systemic sclerosis. Previous reported treatments include diltiazem, bisphosphonates, sodium thiosulfate, and surgical excision (mainstay of treatment) [11, 12].

Further research is needed to understand the link between calcinosis cutis and chronic sclerodermoid GVHD. The recently published prediction model for development of sclerodermoid GVHD suggests an immunological explanation and guides early identification and treatment [8]. This case adds an additional unique cutaneous manifestation of GVHD to the current literature.

## Figures and Tables

**Table 1:** Previous cases of calcinosis cutis associated with sclerodermoid graft versus host disease.

Case	Age^†^	Sex	Comorbidities	Indication for BMT	Time interval^*∗*^	Type and distribution of calcinosis cutis	Biochemistry (calcium and phosphate)
Lipshutz et al. [9]	54	F	T2DM, peripheral neuropathy, diabetic nephropathy, hyperlipidemia, pericarditis, anxiety, and depression	Acute myelogenous leukemia	11 years	Dystrophic Left iliac spines, proximal, and distal lower legs	Calcium (N) Phosphate (low)
Man et al. [10]	60	M	Diabetes, HTN, CAD, gastric ulcers, and pulmonary fibrosis	Acute myelogenous leukemia	N.S	Dystrophic Chest wall, anterior thighs, upper limbs, andlower limbs	Serum calcium (N) Phosphate (N.S)
Current case	59	M	Steroid induced diabetes, pulmonary GVHD, and restrictive lung disease	Follicular non-Hodgkin's lymphoma	12 years	Dystrophic Trunk and bilateral upper limbs	Serum calcium (N) Phosphate (N)

^†^Age in years, F: female, M: male, T2DM: type 2 diabetes mellitus, HTN: hypertension, CAD: coronary artery disease, GVHD: graft versus host disease, ^*∗*^time interval between GVHD and calcinosis cutis, N.S: not stated, N: normal.
